# A five-year review of vertical HIV transmission in a specialized service: cross-sectional study: Revisão de cinco anos da transmissão vertical do HIV em um serviço especializado: estudo transversal

**DOI:** 10.1590/1516-3180.2016.0139140616

**Published:** 2016-11-10

**Authors:** Izabel Cristina Hoffmann, Wendel Mombaque dos Santos, Stela Maris de Mello Padoin, Sonia Maria Oliveira de Barros

**Affiliations:** I PhD. Nurse, University Hospital, Universidade Federal de Santa Maria (UFSM), Santa Maria (RS) Brazil.; II MSc. Nurse, University Hospital, Universidade Federal de Santa Maria (UFSM), Santa Maria (RS), Brazil.; III PhD. Professor, Department of Nursing, Universidade Federal de Santa Maria (UFSM), Santa Maria (RS), Brazil.; IV PhD. Professor, Department of Nursing, Universidade Federal de São Paulo (Unifesp), São Paulo (SP), Brazil.

**Keywords:** HIV, Infectious disease transmission, vertical, Women’s health. Child health, Hospital records, HIV, Transmissão vertical de doença infecciosa, Saúde da mulher, Saúde da criança, Registros hospitalares

## Abstract

**CONTEXT AND OBJECTIVE:**

Healthcare professionals need to instill the process of prevention, control and treatment of people infected with HIV into care practice. Through maintaining preventive treatment among HIV-infected pregnant women, it has been demonstrated that prophylactic antiretroviral therapy, scheduled cesarean section and the prohibition of breastfeeding significantly reduce vertical HIV transmission. This study aimed to assess the rates of vertical HIV transmission in a specialized service and identify the factors associated with it.

**DESIGN AND SETTING:**

Cross-sectional study developed at the University Hospital of Santa Maria (RS), Brazil.

**METHODS:**

A cross-sectional study was conducted on a sample of 198 notification forms and medical records of HIV-positive pregnant women and exposed children.

**RESULTS:**

The vertical transmission rate was 2.4%, and three children had been infected by vertical HIV transmission. The statistically significant risk factor was the use of injectable drugs. Delayed reporting of pregnancy, absence of antiretroviral therapy during pregnancy, lack of proper prenatal care, incapacity to perform viral load detection tests and CD4+ T cell counts and obstetric and maternal clinical complications were reported.

**CONCLUSIONS:**

The vertical transmission rate was high and the recommended intervention measures were not adopted in full. Adequate prophylactic measures need to be implemented in HIV-positive pregnant women prenatally and during the antenatal, delivery and postpartum periods.

## RESUMO

**CONTEXTO E OBJETIVO:**

Os profissionais de saúde precisam introduzir o processo de prevenção, controle e tratamento das pessoas infectadas com HIV na prática assistencial. Manter o tratamento preventivo em gestantes infectadas pelo HIV demonstra que a terapia antirretroviral profilática, a cesariana programada e a proibição da amamentação reduzem significativamente a transmissão vertical do HIV. Este estudo tem como objetivo avaliar as taxas de transmissão vertical do HIV em um serviço especializado e identificar os fatores associados.

**TIPO DE ESTUDO E LOCAL:**

Estudo transversal desenvolvido no Hospital Universitário de Santa Maria (RS), Brasil.

**MÉTODOS:**

Estudo transversal foi conduzido utilizando amostra de 198 fichas de notificação e prontuários de mulheres grávidas HIV-positivas e crianças expostas.

**RESULTADOS:**

A taxa de transmissão vertical foi de 2,4%, e três crianças foram infectadas por transmissão vertical do HIV. O fator de risco estatisticamente significativo foi o uso de drogas injetáveis. Comunicação tardia da gravidez, ausência de terapia antirretroviral durante a gravidez, falta de cuidados pré-natais adequados, incapacidade de realizar testes virais de detecção de carga e contagem de células T CD4+ e complicações clínicas obstétricas e maternas foram relatadas.

**CONCLUSÕES:**

A taxa de transmissão vertical foi elevada e medidas de intervenção recomendadas não foram adotadas na íntegra. Medidas profiláticas adequadas precisam ser implementadas em mulheres grávidas HIV-positivas no pré-natal e durante os períodos pré-natal, intraparto e pós-parto.

## INTRODUCTION

Vertical transmission of human immunodeficiency virus (HIV) is characterized by passage of the virus from the mother to the child through the placenta during pregnancy, at the time of childbirth or through breastfeeding.^1^^,^^2^^,^^3^^,^^4^ Children with HIV infection who develop acquired immunodeficiency syndrome (AIDS) stand out within the context of the epidemic. Its epidemiological growth is due to the feminization process and the increased survival rate among individuals infected through vertical transmission.^5^^,^^6^

In Brazil, the first cases of vertical transmission were reported in 1985.^7^ The Brazilian Ministry of Health uses the incidence rates among children under five years of age as an indicator for monitoring vertical transmission. Between 1980 and 2013, 12,551 cases of AIDS in children under 13 years of age who had been exposed to vertical transmission were reported in the notification information system.^1^

Screening of pregnant women and early implementation of antiretroviral prophylaxis are issues that require more attention.^8^ Healthcare professionals need to instill the process of prevention, control and care of people infected with HIV into general practice. Through administering preventive treatment among HIV-infected pregnant women, it has been demonstrated that prophylactic antiretroviral therapy, scheduled cesarean section and prohibition of breastfeeding significantly reduces HIV transmission from mother to child.^9^^,^^10^

## OBJECTIVE

This study aimed to assess the rate of vertical HIV transmission in a specialized service and identify the factors associated with it.

## METHODS

A cross-sectional study was conducted between 2008 and 2012 at the University Hospital of Santa Maria, Rio Grande do Sul, Brazil, where HIV-infected pregnant women gave birth. The study was approved by the Research Ethics Committee of the Paulista School of Nursing (CAEE No: 16395413.4.0000.5505), in accordance with Resolution 466/2012 of the National Health Council and also with international rules.

The study participants were identified through records kept in the information system for notifiable diseases and through the medical records of HIV-positive women. The sample comprised the entire population of children at this hospital who were exposed to HIV between 2008 and 2012. Miscarriages and cases of stillbirths among pregnant women infected with HIV were excluded.

The primary variable was the prevalence of vertical HIV transmission. Secondary variables included sociodemographic variables (maternal age, maternal education, city of residence, area of residence and occupation of the mother); maternal exposure to HIV (sexual partners, partner’s HIV status and use of injectable drugs); pregnancy, prenatal care, early prenatal care, antiretroviral therapy during pregnancy, treatment regimen, reasons for not performing antiretroviral prophylaxis during pregnancy, maternal HIV viral load (PCR-RNA) and CD4+ T cell counts during pregnancy; complications during pregnancy (clinical, obstetric and coinfections); management during delivery (delivery type, delivery performance of municipality, use of injectable antiretrovirals, rupture of membranes, episiotomy, lactation inhibitors, quick tests before delivery and pregnancy outcome); and finally, care of the newborn (early administration of oral zidovudine, full-time use of oral zidovudine, breastfeeding, cross lactation, viral load, HIV screening test and confirmatory rapid anti-HIV serological test).

Descriptive statistics (absolute frequency, mean, median, standard deviation, minimum and maximum) of the independent variables were performed to characterize the HIV-infected pregnant women and exposed children. The dependent variable was used to calculate the rate of vertical HIV transmission. Fisher’s exact test and nonparametric Mann-Whitney tests were used to assess the associations between the dependent and independent variables.

We used the chi-square test to compare quantitative variables between HIV-positive and HIV-negative children and binary logistic regression to verify the risk factors for vertical HIV transmission. The data were analyzed using the Statistical Package for the Social Sciences (SPSS) 21.0. For each point estimate, 95% confidence intervals were derived, and a statistical significance level of 5% was used.

## RESULTS

Over the study period, there were 198 eligible cases of HIV-infected women who gave birth in the institution. Therefore, 198 children were exposed to HIV during pregnancy. The baseline characteristics of the pregnant women and exposed children are shown in Table 1.

**Table 1.**
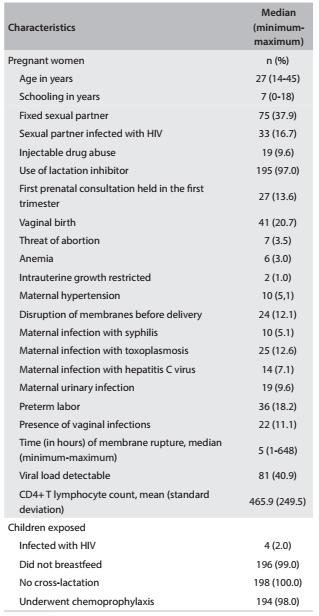
Baseline characteristics of HIV-infected pregnant women and exposed children (n = 198) at the University Hospital of Santa Maria (RS), Brazil, between 2008 and 2012

The prevalence of vertical transmission in this population was 2%, and 98% of the exposed children underwent chemoprophylaxis; 99% were not breastfed.

Among the possible risk factors for HIV transmission, there were significant associations with use of injectable drugs by the pregnant woman, confirmation of HIV infection during prenatal exams, antiretroviral drug use during pregnancy, viral load result, CD4+ T cell counts or presence of associated infections (Table 2).

**Table 2.**
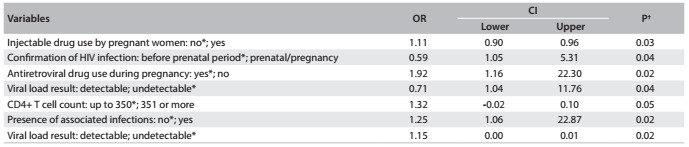
Clinical characteristics of pregnant women infected with HIV at the University Hospital of Santa Maria (RS), Brazil, between 2008 and 2012

*Reference; ^†^Binary logistic regression; OR = odds ratio; CI = confidence interval.

Through assessing the clinical characteristics of the pregnant women infected with HIV, it was found that 39.4% had obstetric complications, 36.4% suffered from coinfections and 30.8% demonstrated presence of maternal clinical complications.

Regarding the number of hours after birth at which antiretroviral therapy was started for the child, a statistically significant difference (P = 0.03) was found between those infected with HIV and those that were not infected.

## DISCUSSION

In Brazil, the prevalence of vertical HIV transmission is 1%. However, the rate seen in the hospital service examined here was 2%, which is more than double the national average. It was observed that this rate was 3.4 times higher than the prevalence in this service, over the period between 2002 and 2006, when it was 0.7%. Some of the factors that may have influenced the increase in vertical transmission are late notification of pregnancies of infected women for the health service, absence of the use of antiretroviral therapy during pregnancy, lack of perception of prenatal complications, obstetric complications and quality of maternal clinics. In 2012, the detection rate for AIDS in children under 5 years of age was 3.4/100,000. This rate has been used as an indicator to monitor vertical HIV transmission.^11^^,^^12^^,^^13^

The present study did not allow identification of risk factors for vertical transmission, mainly because of absence of data in the files. However, there is evidence that clinical and immunological factors (recent infection, vaginal infections, viral load of pregnant women and low CD4+ count), obstetric issues (vaginal delivery and premature birth), factors relating to the newborn (breastfeeding and antiretroviral prophylaxis for newborns), absence of antiretroviral therapy during pregnancy and behavioral factors (young maternal age and use of drugs by pregnant women) influence vertical transmission.^14^

With regard to clinical and immunological factors, it was found in this study that 36.4% of the pregnant women had recent infections and 11.1% had vaginal infections. Both types of infection boost vertical transmission because they weaken immunity in the pregnant woman and fetus and therefore result in greater amounts of secretions with high viral load at the time of delivery.^14^^,^^15^^,^^17^ This demonstrates the importance of early diagnosis and subsequent treatment in order to reduce the risk factors for vertical transmission.

Since the hospital service examined here provides care for cases of high complexity and mainly caters to pregnant women at high risk prenatally, only 22% of the women underwent vaginal delivery and 28.3% gave birth prematurely. The high rate of premature births shown in this study may be associated with the antiretroviral therapy, which has been shown to increase the risk of premature delivery.^9^^,^^10^^,^^12^^,^^14^^,^^16^

With regard to risk factors relating to the newborn, 1.5% of the newborns in this study were breastfed and 97.1% underwent chemoprophylaxis. There is evidence that breastfeeding without antiretroviral treatment for the mother or the child increases the risk of vertical transmission by 91%. One factor that helps reduce the possibility of vertical transmission is the use of medication to inhibit lactation, and 95.9% of the pregnant women in this study were found to be using this medication.^4^^,^^8^^,^^12^^,^^14^^,^^15^^,^^16^^,^^17^^,^^18^

Use of antiretroviral drugs during pregnancy increases the CD4+ T lymphocyte cell counts and consequently reduces the viral load, thereby decreasing the risk of intrauterine vertical transmission. This should be combined with early detection of HIV infection in pregnant women. However, 38.3% of the women in this study were diagnosed with infection prenatally or later. Only 33.3% of the pregnant women received adequate prenatal care starting from the first trimester of pregnancy, and this lack of care can contribute towards the risk of vertical transmission, abortion and perinatal mortality.^9^^,^^10^^,^^15^^,^^16^^,^^17^

Injectable drug abuse, which doubles the risk of vertical transmission and increases the risk of abortion sixfold,^10^ was seen in the cases of 9.6% of the pregnant women in this study. For pregnant women who use other drugs, harm reduction strategies should be discussed. It needs to be emphasized that they should use condoms and not share syringes and needles, if they are using injectable drugs. In this manner, users can reduce their own risk of reinfection and transmission to their sexual partners.^19^

The diagnosis of vertical transmission needs to be confirmed by performing a screening test for HIV-1 and HIV-2 and at least one confirmatory test. In the event of positive results, a new sample should be collected to confirm the positive results from the first sample.^3^^,^^4^^,^^13^

The cases of 81.1% of the HIV-positive pregnant women were only notified in the third trimester, thus indicating that there were delays in notification. In Brazil, notification is supposed to occur at the time of detection of HIV infection in pregnant women and/or children infected by vertical transmission.^4^

Healthcare professionals need to work towards reducing vertical HIV transmission through providing recommendations for interventions, early identification of women exposed to HIV, implementation of preventive measures and appropriate use of antiretroviral therapy in prenatal care. Controlling AIDS can prove to be a challenge.^8^

The limitations of this study included its cross-sectional design, which did not allow causality to be established. Moreover, the primary source of data was the notification forms and medical records of the pregnant women and children exposed to HIV.

## CONCLUSION

This study showed that the rate of vertical transmission in a specialized service is 2%, twice the national average. Risk factors for increased vertical transmission of HIV were: injected drug use, confirmation of HIV infection in prenatal period or childbirth, non-use of antiretroviral drugs during pregnancy, high viral load, reduced CD4 T lymphocytes and presence of associated infections.
